# Exploring the baseline cardiac function and its correlation with risk stratification in children diagnosed with acute lymphoblastic leukemia

**DOI:** 10.1007/s00277-025-06523-7

**Published:** 2025-07-30

**Authors:** Diana R. Lazar, Dana Maniu, Florin-Leontin Lazar, Cristina Blag, Madalina Bota, Mihnea Zdrenghea, Simona Cainap

**Affiliations:** 1 Department No. 10, Oncology, “Iuliu Hatieganu” University of Medicine and Pharmacy, Cluj-Napoca, 400012 Romania; 2 Department of Pediatric Cardiology, Emergency Clinical Hospital for Children, Cluj-Napoca, 400394 Romania; 3 Biomolecular Physics Department, Faculty of Physics, “Babes-Bolyai” University, Cluj-Napoca, 400084 Romania; 4 Department No. 4, Internal Medicine, Medical Clinic Number 1, “Iuliu Hatieganu” University of Medicine and Pharmacy, Cluj-Napoca, 400012 Romania; 5 Department of Hematology, Emergency Clinical Hospital for Children, Cluj-Napoca, 400394 Romania; 6 Department of Mother and Child, “Iuliu Hatieganu” University of Medicine and Pharmacy, Cluj-Napoca, 400012 Romania; 7 Department of Hematology, “Ion Chiricuta” Oncology Institute, Cluj-Napoca, 400015 Romania

**Keywords:** Childhood acute lymphoblastic leukemia, Pre-chemotherapy, Cardiac dysfunction, Cardiac biomarkers, Echocardiography

## Abstract

Childhood leukemia survivors are at a lifelong risk of cardiac dysfunction due to anthracycline treatment. This study is among the first to assess pre-chemotherapy cardiac abnormalities in children newly diagnosed with acute lymphoblastic leukemia (ALL), aiming to identify early indicators of cardiac dysfunction before anthracycline exposure. Existing literature largely focuses on cardiac damage following chemotherapy, but our findings suggest that subclinical cardiac changes may already be present at diagnosis. The cohort included 47 children diagnosed with ALL, none of whom presented clinical signs of cardiac disease. A baseline cardiac evaluation was performed before the onset of chemotherapy. Our findings demonstrated that 29.78% of patients had elevated hs-Troponin and 44.68% had elevated NT-proBNP levels before chemotherapy, with patients in the high relapse risk group (HRG) presenting significantly increased rates of abnormal values. Also, ECG abnormalities, including abnormal heart rate and PR interval, were more frequent in this group. Echocardiography revealed higher LV mass index and more diastolic dysfunction in the HRG compared to the intermediate-risk group. What is more, female patients showed greater baseline cardiac vulnerability, and younger children had proportionally higher indexed LV mass despite lower absolute LV mass. These results suggest that cancer itself may influence cardiac function before chemotherapy, potentially predisposing pediatric patients to long-term cardiac dysfunction.

**Supplementary Information:**

The online version contains supplementary material available at 10.1007/s00277-025-06523-7.

## Introduction

Childhood leukemia survivors present a lifelong increased risk of developing cardiac dysfunction following anthracycline (AC) treatment. This well-known risk has prompted ongoing research into the long-term effects of AC on heart function. However, recent studies have shifted focus to investigate the potential impact cancer itself has on cardiac function, even before the onset of chemotherapy. More exactly, cardiac function in patients with an active cancer could be influenced by various factors, including the pro-inflammatory state induced by malignancy [1], along with the metabolic and neuro-hormonal disturbances that accompany the disease [2]. Inflammatory cytokines, well known to be elevated in the oncologic patient, can cause altered vascular tone, increase myocardial wall stress, and cause myocardial contractile dysfunction by altering intracellular calcium transport [3, 4]. Additionally, neuro-hormonal imbalance observed in cancer patients, such as increased catecholamine levels or renin-angiotensin-aldosterone system activation, can further contribute to the development of cardiac dysfunction over time [2, 5]. What is more, in patients with leukemia, leukemic infiltration of the myocardium could further impact cardiac function [6, 7].

In addition to these factors, it is important to note that in the adult population, cancer and heart disease share several common risk factors. Smoking, diabetes, hypertension, and obesity are all risk factors that can increase the likelihood of both malignancy and cardiac disease. The systemic metabolic alterations associated with these risk factors could create a bidirectional relationship between the two conditions, with one exacerbating the other. However, pediatric cancers, including childhood leukemia, are less frequently associated with the traditional metabolic risk factors seen in adults. Instead, childhood malignancies risk factors are more likely to be genetic and environmental [8].

Acute lymphoblastic leukemia (ALL) is the most frequently encountered pediatric cancer. Over the past two decades, overall survival in pediatric ALL has significantly improved, with current 5-year survival rates exceeding 90% in many high-income settings, largely due to risk-adapted therapy, precise minimal residual disease monitoring, and international collaborative trials [9–12].

Anthracyclines, especially doxorubicin and daunorubicin, are vital in pediatric ALL treatment but carry a significant risk of dose-related cardiotoxicity, which can lead to long-term heart problems such as cardiomyopathy and heart failure. Dexrazoxane has been proven to effectively reduce this risk without compromising treatment effectiveness and is increasingly used along with routine cardiac monitoring. Other agents like cyclophosphamide and immunotherapies (e.g., CAR T-cells) may also cause temporary or rare heart effects, highlighting the importance of long-term cardiac monitoring in ALL survivors [13, 14].

Early recognition of cardiac alterations in pediatric ALL patients is essential for identifying those at elevated risk of developing cardiotoxicity, particularly due to cumulative anthracycline exposure. Recent studies highlight that subclinical cardiac dysfunction can occur even at lower doses and often precedes overt symptoms. Tools such as speckle-tracking echocardiography and biomarkers like troponin and NT-proBNP have improved the early detection of myocardial injury, enabling timely risk stratification [15, 16].

Identifying high-risk patients early allows for the prompt initiation of cardioprotective strategies, such as dexrazoxane, which has been shown to preserve left ventricular function without compromising event-free or overall survival [17, 18].

Additionally, early detection supports personalized surveillance protocols, where high-risk patients receive more frequent cardiac monitoring and long-term follow-up, in line with recent Children’s Oncology Group (COG) survivorship guidelines [19].

This proactive approach not only reduces the incidence of chronic cardiac disease in survivors but also facilitates informed decisions about modifying chemotherapeutic regimens when necessary, balancing oncologic efficacy with long-term health outcomes.

The present study, therefore, focused on evaluating baseline cardiac function in pediatric patients diagnosed with ALL. The primary goal was to distinguish any early, pre-treatment modification in cardiac function that could serve as early indicators of myocardial dysfunction. By identifying these changes in the initial phases of chemotherapy, healthcare providers could better predict the likelihood of AC-induced cardiac toxicity and tailor treatment plans accordingly to mitigate the risk of long-term cardiac damage. Such early detection could ultimately serve as a blueprint for understanding and managing chemotherapy-induced cardiotoxicity in pediatric patients, potentially improving long-term cardiovascular outcomes for survivors of childhood leukemia.

## Materials and methods

This cross-sectional study included pediatric patients diagnosed with ALL from a single hemato-oncology center in Cluj-Napoca, Romania. Patients were enrolled from June 2019 to December 2024. All children were diagnosed and treated according to the ALL-IC BFM 2009 protocol [13]. After completing the first chemotherapy phase (protocol IA), patients are assigned to three ALL risk groups: standard (SRG), intermediate (IRG), and high (HRG). Patients in the HRG undertake a more intensive chemotherapy regimen. Exclusion criteria were pre-existing heart disease, anterior thoracic irradiation, or anterior chemotherapy. Patients in the SRG were also excluded from the statistical analysis, since the initial lot had only one patient in this group. Written informed consent was obtained from all participants/parents and/or legal guardians. The study protocol was approved by the University of Medicine and Pharmacy “Iuliu Hatieganu” Clinical Research Ethics Committee.

Before starting chemotherapy, all patients were assessed for potential risk factors for cardiotoxicity, as described in the article on cardiotoxicity in children with cancer by Bertorello N et al. [20]. According to their paper, children under the age of 10, females, presence of specific genetic factors (such as trisomy 21, ataxia, or other polymorphic gene variations in AC metabolism), and coexisting cardiovascular diseases are associated with an increased risk of doxorubicin-induced cardiac toxicity. Naturally, treatment-related factors further increase this risk, including total AC dose exceeding 250 mg/m2 and concurrent therapies with CPM or other chemotherapies with cardiotoxic potential.

All patients received baseline cardiologic evaluation as follows: (i) assessment of serum high-sensitivity (hs) Troponin and NT-proBNP; (ii) trans-thoracic echocardiography (TTE) with pulsed wave Doppler and TDI evaluation; (iii) bedside ECG; (iv) 24-hour 12-lead Holter ECG evaluation. Left ventricle (LV) mass, left ventricle mass index (LVMI), and relative wall thickness (RWT) were calculated according to European Society of Cardiology (ESC) guidelines [6]. Normal values were interpreted according to pediatric nomograms, with abnormal values considered above the 98th or below the 2nd percentile for age and sex [21, 22].

Baseline echocardiographic parameters were assessed using a VIVID S5 echocardiograph. Heart function was evaluated in the parasternal long axis and the apical four-chamber view. Left ventricular systolic function was assessed by calculating the left ventricular ejection fraction (LVEF) and shortening fraction (LVSF) using two methods: M-mode and the volumetric method. To assess diastolic function, we used both mitral inflow pulsed-wave Doppler (early diastole - E, and late diastole - A), as well as TDI (early diastole– e’, and late diastole– A’). Diastolic function was considered to be normal for E/A values between 2.3 ± 0.6 and E/e’ below 10, pseudo-normal for similar E/A values but E/e’ above 10, abnormal relaxation when E/A values were below 1 with E/e’ below 10, and restrictive pattern when E/A values were above 1.5 with E/e’ above 10 [23].

The primary endpoint of this study was to detect any sign of abnormal cardiac function before the administration of any cardiotoxic agent. Secondly, a comparison of cardiac function between patients in the IRG and HRG was done. What is more, possible patient-related risk factors for cardiac toxicity were assessed. Lastly, an evaluation of cardiac function parameters was done between patients with B-cell and T-cell ALL.

Statistical analysis was done using Microsoft Excel 2019. To assess differences encountered between the IRG and HRG groups, continuous variables were analyzed using the two-sample t-test. The normally distributed continuous data were presented as mean ± standard deviation (SD). Qualitative variables were described as numbers (n) and percentages (%). The statistical significance of the obtained results was determined using the corresponding two-tailed p-value. Results with a p-value less than 0.05 were considered statistically significant. The Pearson correlation coefficient was calculated to evaluate possible correlations.

## Results

The study enrolled a total of 47 consecutive children diagnosed with ALL, mostly boys (53%), with a mean age at diagnosis of 5.85 ± 3.45. Regarding the distribution between the afore-mentioned ALL risk groups, there were 29 patients in the IRG group and 17 patients in the HRG group, while only one patient remained in the SRG group. Table 1 summarizes the main patient characteristics. The patient in the SRG group was excluded from statistical analysis due to the insufficient sample size (*n* = 1), which prevented any meaningful statistical comparison.

**Table 1:** Patient characteristics according to ALL risk group stratification

Characteristics	*n* (%)	IRG (*n*)	HRG (*n*)	SRG (*n*)
Gender				
female	22 (46.81%)	14	7	1
male	25 (53.19%)	15	10	0
Age				
1–3 years	11 (23.40%)	6	4	1
3–6 years	19 (40.43%)	14	5	0
6–10 years	10 (21.82%)	7	3	0
10–15 years	5 (10.64%)	2	3	0
15–18 years	2 (4.26%)	0	2	0
Origin				
urban	20 (42.55%)	13	7	0
rural	27 (57.45%)	16	10	1
ALL type				
B cell ALL	38 (80.85%)	24	13	1
T cell ALL	9 (19.15%)	5	4	0
BMI				
underweight	15 (31.91%)	9	5	1
normal	20 (42.55%)	11	9	0
overweight	5 (10.64%)	4	1	0
obesity	7 (14.89%)	5	2	0
severe obesity	0 (0.00%)	0	0	0

All patients underwent cardiologic evaluation before the start of chemotherapy, and none demonstrated clinical signs or symptoms indicative of cardiac disease at baseline. The primary findings concerning pre-chemotherapy cardiac function according to ALL relapse risk group, are summarized in Table 2.

**Table 2:** Comparison of baseline cardiac biomarkers and ECG parameters between relapse risk groups in children with ALL

	IRG (*n* = 29)	HRG (*n* = 17)	*p*-value
Biologic*			
Elevated hs-Troponin	5 (17.24%)	8 (47.06%)	***0.030***
Elevated NT-proBNP	10 (34.48%)	11 (64.71%)	***0.047***
ECG**			
Abnormal HR	7(24.14%)	9 (52.94%)	***0.048***
Abnormal PR interval	3 (10.34%)	6 (35.29%)	***0.040***
HRV parameters***			
SDNN (ms)	167.40 ± 44.49	149.64 ± 45.93	0.296
SDANN (ms)	130.29 ± 37.93	124.64 ± 41.21	0.672
RMSSD (ms)	110.71 ± 48.81	99.36 ± 45.48	0.459
pNN50 (%)	39.12 ± 16.60	30.80 ± 16.40	0.142
LF/HF	0.66 ± 0.24	0.63 ± 0.11	0.698
Echocardiography– M mode			
LV mass (g)	50.40 ± 25.00	45.97 ± 28.42	0.644
LVMI (g/m2)	68.01 ± 29.18	48.45 ± 23.71	***0.045***
RWT (cm)	0.33 ± 0.08	0.27 ± 0.15	0.186
LVEF (%)	66.47 ± 5.72	64.21 ± 12.25	0.499
LVSF (%)	36.33 ± 4.77	37.45 ± 7.33	0.634
Echocardiography– TDI– Diastolic function			
Normal	26 (90%)	14 (83%)	0.478
Restrictive	3 (10%)	3 (16.67%)	0.478

Data were shown as n (%) or mean ± SD; p-value under 0.05 was considered to be statistically significant; * Troponin and NT-proBNP values over the 97.5 percentile for age and sex were considered to be elevated; ** HR and PR interval values above the 98th or below the 2nd percentile for age and sex were considered to be abnormal; *ALL* acute lymphoblastic leukemia, *HR* heart rate; *** SDNN standard deviation of all normal sinus RR intervals over 24 h, *SDANN* standard deviation of the average normal sinus RR intervals for all 5 min segments over 24 h, *rMSSD* root mean square of the successive normal sinus RR interval difference, *pNN50* percentage of successive normal sinus RR intervals over 50 milliseconds, *LF* low frequency, *HF* high frequency

To begin with, abnormally high levels, for age and sex, of hs-Troponin and NT-proBNP were identified in 29.78% and 44.68% of patients, respectively. Also, a strong positive correlation (Pearson coefficient 0.843) of NT-proBNP levels and leucocyte count at diagnosis was identified. What is more, a significantly higher percentage of patients in the HRG had hs-Troponin and NT-proBNP values outside the normal range for age and sex, as compared to patients in the IRG.

When evaluating bedside ECG, heart rate (HR) values above the upper normal limit for age and sex were noticed in 23.91% of patients, and values below the lower normal limit for age and sex in 10.86% of patients. Significantly, more patients in the HRG presented with HR values outside the normal range for age and sex than the patients in the IRG. Also, significantly more abnormal PR interval values were identified in patients belonging to the HRG as compared to the IRG. Regarding the 24-hour Holter ECG readings, heart rate variability (HRV) parameters were slightly lower in the HRG, although not statistically significant.

What is more, the echocardiographic evaluation indicated that despite similar LVEF and LVSF, there was a statistically significant difference between the two compared groups in LVMI. When evaluating diastolic function, we found that more than half of our patients had E/A values outside the normal range for age, while only 33.33% of patients showed E/e’ values outside the normal range [23]. When comparing patients by risk groups, 66.67% and 50% of patients in the HRG had E/A and E/e’ values outside the normal range for age, whereas only 50% and 20% of patients in the IRG showed abnormal values. However, the difference between the two groups was not statistically significant (*p* = 0.358). We also identified a total of 6 patients with a restrictive filling pattern, with no significant difference between the risk groups. No patients exhibited abnormal filling patterns, and only one patient from the SRG showed a pseudo-normal filling pattern. Since there was only one SRG patient, we could not perform a statistical analysis of this finding.

Next, we analyzed the same cardiac function parameters between patients with B-cell ALL and patients with T-cell ALL (Supplementary information - Table S1). Patients with T-cell ALL presented statistically significantly higher values for several HRV parameters, such as RMSSD (*p* = 0.001), pNN50 (*p* = 0.012), and a lower LF/HF ratio (*p* < 0.001). No additional statistically significant differences were observed.

We also analyzed baseline cardiac function parameters in relation to patient-specific cardiotoxicity risk factors (Supplementary information - Table S2). Our results indicated that female sex was associated with significantly more frequent elevated hs-Troponin values, as compared to males in our cohort (*p* = 0.018). Also, abnormal QTc interval values were significantly more common in females than in males (*p* = 0.003). What is more, LVEF was significantly lower in females compared to males (*p* = 0.047). Regarding age-based differences, PR interval abnormalities were significantly more frequent in children above 10 years old compared to those under 10 (*p* < 0.001). QTc prolongation was more frequent in children under 10 years old, as compared to those above 10 years old (*p* = 0.017). LV mass was significantly higher in the older group (*p* = 0.017), while LVMI was significantly higher in younger children (*p* = 0.025). No other statistically significant differences were noticed.

## Discussions

To our knowledge, this is one of the first studies to investigate pre-chemotherapy cardiac abnormalities in children diagnosed with ALL. Most studies investigating cardiac function in children with leukemia have evaluated cardiac function after AC treatment. Indeed, such studies have consistently shown an increased likelihood of developing diastolic dysfunction years after finishing the oncologic treatment. Unfortunately, diastolic dysfunction frequently presents before any overt clinical symptoms arise or before significant reductions in LVEF are detected. Studies have shown that by the time reductions in LVEF are apparent, significant structural and functional cardiac changes may have already occurred, thereby highlighting the importance of early intervention [24, 25].

This study aims to fill a gap in the literature by assessing cardiac function in pediatric ALL patients before starting chemotherapy. By detecting cardiac abnormalities early, it hopes to determine whether pre-existing cardiac alterations increase the risk of future cardiac complications after treatment. Additionally, understanding cardiac function before treatment could help clinicians develop more targeted surveillance strategies, allowing for early detection of heart dysfunction and the implementation of preventive measures. These steps could improve long-term cardiovascular outcomes for this vulnerable group.

In their study, Bertorello et al. [20] identified several key risk factors for cardiotoxicity. However, in our cohort, only patient-related risk factors could be assessed, as no chemotherapy had been administered prior to the cardiac evaluation. Despite this limitation, our findings reveal important differences in cardiac function based on sex and age, underscoring the potential relevance of these factors even before the onset of treatment.

Our findings suggest that female pediatric patients may be at greater risk of subclinical cardiac involvement even before chemotherapy begins. The higher frequency of elevated troponin and QTc prolongation, combined with a lower LVEF, could indicate a greater baseline vulnerability to cardiotoxicity. This aligns with current knowledge in pediatric cardio-oncology [26]. However, reports from adult cardio-oncology are contradictory, with some supporting the hypothesis that females may be more vulnerable to cardiotoxicity, while others deny it [27, 28].

What is more, age differences observed in our cohort indicate both differences in physiology between younger (under 10 years old) and older (above 10 years old) children, as well as possible age-related vulnerability in the younger group. As such, we noticed that conduction abnormalities (PR interval variations) were more common in older children, which may reflect an increased vagal tone. Higher LV mass in older children is expected due to growth, but when indexed to BSA, the LVMI was higher in younger children, suggesting proportionally greater myocardial mass relative to body size. QTc prolongation, however, was more common in younger children, potentially due to developmental differences in ventricular repolarization or heightened sympathetic activity in this age group. Nevertheless, these results emphasize the importance of age-specific reference ranges when interpreting pediatric cardiac parameters and suggest that age-related physiological changes may influence baseline vulnerability to cardiac dysfunction.

To date, several studies have investigated cardiac function in adult cancer patients before starting chemotherapy. Notably, Tadic et al. evaluated 122 adult patients with solid tumors, along with 45 controls matched for age and sex. Their results clearly showed that patients with cancer had a significantly reduced myocardial strain (including circumferential, radial, and longitudinal measures) [29]. Another study by Assuncao et al. examined 76 adult patients with acute leukemia and compared them to 76 matched controls based on age, sex, and cardiovascular risk factors. Similar to the previous study, they found that global longitudinal strain (GLS) was decreased, while left ventricular mass (LVM) was increased in patients with AL compared to controls [6]. Lastly, Fabiani et al. reviewed recent literature on subclinical cardiac damage in cancer patients before undergoing chemotherapy. They concluded that these patients should be considered at risk for developing overt heart failure (HF) and subclinical HF, warranting inclusion in primary preventive strategies even before chemotherapy begins [2].

Although we did not evaluate cardiac function in our patients using GLS, we observed that a higher percentage of our patients had TDI parameter values outside the normal range for their age, primarily among those in the high-risk group (HRG). Additionally, six patients displayed a restrictive filling pattern, despite having no prior known risk factors for cardiac disease before their cancer diagnosis. The difference in LVMI between the two groups also indicates a slight myocardial remodeling in early ALL, which is significant because remodeling often precedes alterations in LVEF. These findings suggest that cardiac function can indeed be impacted by cancer itself, highlighting the importance of better risk stratification for cardiac disease in pediatric oncology patients right from diagnosis. Apart from GLS evaluation, recent studies and guidelines, including the ESC cardio-oncology guidelines, highlight cardiac magnetic resonance as an effective method of evaluating global cardiac function (ESC 2022). However, we could not perform cMRI on our patients, because of limited financial and human resources in our center. Also, to obtain adequate cardiac imaging, children would have needed general anesthesia. This would have been an unnecessary added risk to their already complex medical state.

Regarding cardiac biomarkers, our research has shown that these specific markers increase in pediatric cancer patients even before starting chemotherapy, with NT-proBNP proving to be a more sensitive indicator of heart function changes. This effect is even more pronounced in the HRG, which can be attributed to the fact that children in this group are more likely to have myocardial infiltration, or a higher leukocyte count at diagnosis. As several studies have demonstrated, leukemic infiltration of the myocardium appears to be directly proportional to the levels of circulating leukemic cells [30–32]. Indeed, our results show a strong positive correlation between NT-proBNP levels at diagnosis and leukemic count, further supporting this hypothesis. However, NT-proBNP levels are known to reflect LV volume or pressure overload. Children being treated for ALL often require intensive intravenous fluid administration to prevent life-threatening consequences of tumor lysis syndrome. In a recent study, Pawlik et al. detected an increase in NT-proBNP levels without a significant change in Troponin concentration 24 h after starting fluid therapy in children with hematological malignancies [33]. These intensive fluid regimens may be responsible for the increased levels observed.

Autonomous nervous system (ANS) dysfunction is common in patients with advanced cancers and has been shown to increase the risk of adverse cardiovascular outcomes [34]. However, the exact implications of ANS dysfunction in cancer patients are still not fully understood. Additionally, the stress related to cancer diagnosis and treatment can enhance sympathetic nervous activity while reducing parasympathetic tone, leading to a decrease in HRV parameters. Our preliminary ECG data show a slight decrease in HRV among patients in the HRG, which, although not statistically significant, suggests the possibility of early cardiac ANS dysfunction in these patients. This is also reflected in the difference in HR between the two groups, with HRG patients exhibiting more abnormal HR values. In another study, our team observed a decrease in HRV parameters in children treated for leukemia after receiving half of the total cumulative doxorubicin dose [35]. A review by Kloter et al. demonstrated that higher HRV is associated with a better prognosis and slower cancer progression [36]. These findings highlight the impact of cancer and its treatment on normal cardiac function and underscore the importance of better understanding their implications and prognostic value in children with leukemia.

A few significant differences in baseline cardiac function were noted between B-cell and T-cell ALL patients, particularly in HRV parameters. T-cell ALL patients had higher RMSSD and pNN50 values and a lower LF/HF ratio, indicating more dominant parasympathetic tone and better preserved autonomic cardiac function. This may reflect underlying differences in disease biology, inflammatory responses, or autonomic regulation between the two subtypes.

Although our study had a relatively small sample size, the incidence of pediatric leukemia is 34 cases per 1 million [21], so our results are representative. Notably, this study is among the first to investigate pre-chemotherapy cardiac abnormalities in pediatric ALL patients, shedding light on an important yet underexplored area of research, despite the limitation of a small sample size. However, larger studies are needed to confirm our findings. Additionally, comparing with an age- and sex-matched control group would more accurately highlight the differences in cardiac function between healthy children and those with ALL.

## Conclusions

This study revealed early alterations in cardiac function among pediatric patients newly diagnosed with ALL, even before the initiation of chemotherapy. Notably, patients in the HRG exhibited more pronounced changes in cardiac parameters compared to others. Given that these patients typically undergo more intensive treatment regimens, these baseline differences may represent a form of preconditioning for subsequent cardiac injury. Our findings suggest that cardiac follow-up protocols could be tailored according to each patient’s relapse risk group to help prevent long-term cardiac damage. Additionally, our results demonstrated that sex and age significantly impact baseline cardiac function, emphasizing the importance of incorporating age- and sex-specific assessments into pre-treatment evaluations to identify those at higher risk of cardiotoxicity. Finally, these preliminary observations highlight the complex interplay between leukemia and cardiac function, pointing to the need for further investigation into the underlying molecular and neurohormonal mechanisms linking the two.

## Supplementary Information

Below is the link to the electronic supplementary material.

Supplementary Material 1

## Data Availability

No datasets were generated or analysed during the current study.

## Abbreviations

AC: anthracycline
AL: acute leukemia
ALL: acute lymphoblastic leukemia
ECG: electrocardiography
GLS: global longitudinal strain
HRG: high-risk group
HRV: heart rate variability
LVEF: left ventricular ejection fraction
LVSF: left ventricular shortening fraction
LVMI: left ventricle mass index
IRG: intermediate risk group
SRG: standard risk group
SDNN: standard deviation of all normal sinus RR intervals over 24 h
SDANN: standard deviation of the average normal sinus RR intervals for all 5 min segments over 24 h
RMSSD: root mean square of the successive normal sinus RR interval difference
pNN50: percentage of successive normal sinus RR intervals over 50 milliseconds
LF: low frequency
HF: high frequency
TDI: tissue Doppler imaging
