# Supplementary material for: Exploring the baseline cardiac function and its correlation with risk stratification in children diagnosed with acute lymphoblastic leukemia
Source: Ann Hematol. 2025 Jul 30;104(8):4157–64. doi: 10.1007/s00277-025-06523-7 (PMC12432049; doi:10.1007/s00277-025-06523-7)
Supplement: Supplementary file 1 — Supplementary Material 1 [file 277_2025_6523_MOESM1_ESM.docx]

**Exploring the baseline cardiac function and its correlation with risk stratification in children diagnosed with acute lymphoblastic leukemia**

Diana R. Lazar^1,2^, Dana Maniu^3*^, Florin-Leontin Lazar^4^, Cristina Blag^5,6^, Madalina Bota^5,6^, Mihnea Zdrenghea^1,7^, Simona Cainap ^2,6^

^1^Department No. 10, Oncology, “Iuliu Hatieganu” University of Medicine and Pharmacy, 400012 Cluj-Napoca, Romania;

^2^Department of Pediatric Cardiology, Emergency Clinical Hospital for Children, 400394 Cluj-Napoca, Romania;

^3^Biomolecular Physics Department, Faculty of Physics, “Babes-Bolyai” University, 400084 Cluj-Napoca, Romania

^4^Department No. 4, Internal Medicine, Medical Clinic Number 1, “Iuliu Hatieganu” University of Medicine and Pharmacy, 400012 Cluj-Napoca, Romania

^5^Department of Hematology, Emergency Clinical Hospital for Children, 400394 Cluj-Napoca, Romania

^6^Department of Mother and Child, “Iuliu Hatieganu” University of Medicine and Pharmacy, 400012 Cluj-Napoca, Romania

^7^Department of Hematology, “Ion Chiricuta” Oncology Institute, 400015 Cluj-Napoca, Romania

**Table S1.** Comparison of baseline cardiac function between patients with B-cell ALL and patients with T-cell ALL.

|  | **B-cell ALL** | **T-cell ALL** | **p- value** |
| --- | --- | --- | --- |
| **Biologic*** | | | |
| **Elevated hs-Troponin** | 7 (18.42%) | 3 (33.33%) | 0.326 |
| **Elevated NT-proBNP** | 19 (50.00%) | 4 (44.44%) | 0.764 |
| **ECG parameters**** | | | |
| **Abnormal HR** | 12 (31.58%) | 4 (44.44%) | 0.464 |
| **Abnormal PRi** | 6 (15.79%) | 2 (22.22%) | 0.644 |
| **Abnormal QTc** | 11 (28.95%) | 1 (11.11%) | 0.270 |
| **SDNN (ms)** | 158.19±43.83 | 188.67±45.94 | 0.212 |
| **SDANN (ms)** | 128.13±37.04 | 138.50±44.12 | 0.636 |
| **RMSSD (ms)** | 101.26±47.11 | 152.83±21.65 | ***0.001*** |
| **pNN50 (%)** | 36.28±18.82 | 47.36±5.12 | ***0.012*** |
| **LF/HF** | 0.69±0.20 | 0.47±0.08 | ***<0.001*** |
| **Echocardiographic parameters** | | | |
| **Normal TDI pattern** | 34 (89.47%) | 9 (100.00%) | 0.309 |
| **Restrictive TDI pattern** | 4 (10.53%) | 0 (0.00%) |  |
| **LV mass (g)** | 52.15±23.68 | 56.98±14.88 | 0.598 |
| **LVMI (g/m^2^)** | 64.7±22.77 | 70.5±20.53 | 0.624 |
| **RWT (cm)** | 0.34±0.08 | 0.33±0.02 | 0.517 |
| **LVEF (%)** | 65.07±8.75 | 68.17±9.87 | 0.535 |
| **LVSF (%)** | 36.50±4.75 | 37.83±8.74 | 0.753 |

Data were shown as n (%) or mean ± SD; p-value under 0.05 was considered to be statistically significant; * Troponin and NT-proBNP values over the 97.5 percentile for age and sex were considered to be elevated; ** HR and PR interval values above the 98^th^ or below the 2^nd^ percentile for age and sex were considered to be abnormal; ALL = acute lymphoblastic leukemia; HR = heart rate; SDNN = standard deviation of all normal sinus RR intervals over 24 h; SDANN = standard deviation of the average normal sinus RR intervals for all 5 min segments over 24 h; rMSSD = root mean square of the successive normal sinus RR interval difference; pNN50 = percentage of successive normal sinus RR intervals over 50 milliseconds; LF = low frequency; HF = high frequency.

**Table S2.** Correlation between baseline cardiac function and patient-related cardiac risk factors (age, sex).

|  | **Female** | **Male** | **p -value** | **Age** | | **p -value** |
| --- | --- | --- | --- | --- | --- | --- |
|  |  |  |  | **<10 years old (n=40)** | **≥ 10 years old (n=7)** |  |
| **Biologic*** | | | | | | |
| **Elevated hs-Troponin** | 8 (36.36%) | 2 (8.00%) | ***0.018*** | 8 (20.00%) | 2 (28.57%) | 0.609 |
| **Elevated NT-proBNP** | 14 (63.64%) | 9 (36.00%) | ***0.059*** | 19 (47.50%) | 4 (57.14%) | 0.638 |
| **ECG parameters**** | | | | | | |
| **Abnormal HR** | 8 (36.36%) | 8 (32.00%) | 0.753 | 13 (32.50%) | 3 (42.86%) | 0.186 |
| **Abnormal PRi** | 4 (18.18%) | 4 (16.00%) | 0.843 | 4 (10.00%) | 4 (57.14%) | ***<0.001*** |
| **Abnormal QTc** | 10 (45.45%) | 2 (8.00%) | ***0.003*** | 11 (27.50%) | 1 (14.29%) | ***0.017*** |
| **SDNN (ms)** | 149.65±42.39 | 174.60±45.07 | 0.101 | 162.76±45.7 | 166.25±44.53 | 0.904 |
| **SDANN (ms)** | 120.71±35.64 | 137.55±39.09 | 0.191 | 127.73±38.21 | 147±36.27 | 0.435 |
| **RMSSD (ms)** | 102.29±50.10 | 115.85±45.06 | 0.410 | 111.97±48.75 | 90.25±34.83 | 0.375 |
| **pNN50 (%)** | 36.23±20.48 | 39.65±15.03 | 0.584 | 38.88±17.29 | 31.47±20.54 | 0.583 |
| **LF/HF** | 0.70±0.26 | 0.61±0.12 | 0.230 | 0.65±0.21 | 0.67±0.13 | 0.828 |
| **Echocardiographic parameters** | | | | | | |
| **Normal TDI pattern** | 19 (86.36%) | 24 (96%) | 0.237 | 36 (90.00%) | 7 (100.00%) | 0.382 |
| **Restrictive TDI pattern** | 3 (13.64%) | 1 (4.00%) |  | 4 (10.00%) | 0 (0.00%) |  |
| **LV mass (g)** | 47.14±24.09 | 59.44±18.74 | 0.127 | 48.38±20.65 | 77.33±16.11 | ***0.017*** |
| **LVMI (g/m^2^)** | 62.64±26.5 | 68.96±16.31 | 0.432 | 67.94±23.57 | 53±7.89 | ***0.025*** |
| **RWT (cm)** | 0.32±0.09 | 0.35±0.04 | 0.271 | 0.33±0.08 | 0.36±0.04 | 0.237 |
| **LVEF (%)** | 62.72±9.16 | 68.88±7.70 | ***0.047*** | 64.64±9.32 | 70.17±5.7 | 0.105 |
| **LVSF (%)** | 34.87±4.49 | 38.67±6.32 | 0.078 | 36±5.79 | 39.83±4.74 | 0.152 |

Data were shown as n (%) or mean ± SD; p-value under 0.05 was considered to be statistically significant; * Troponin and NT-proBNP values over the 97.5 percentile for age and sex were considered to be elevated; ** HR and PR interval values above the 98^th^ or below the 2^nd^ percentile for age and sex were considered to be abnormal; ALL = acute lymphoblastic leukemia; HR = heart rate; SDNN = standard deviation of all normal sinus RR intervals over 24 h; SDANN = standard deviation of the average normal sinus RR intervals for all 5 min segments over 24 h; rMSSD = root mean square of the successive normal sinus RR interval difference; pNN50 = percentage of successive normal sinus RR intervals over 50 milliseconds; LF = low frequency; HF = high frequency.


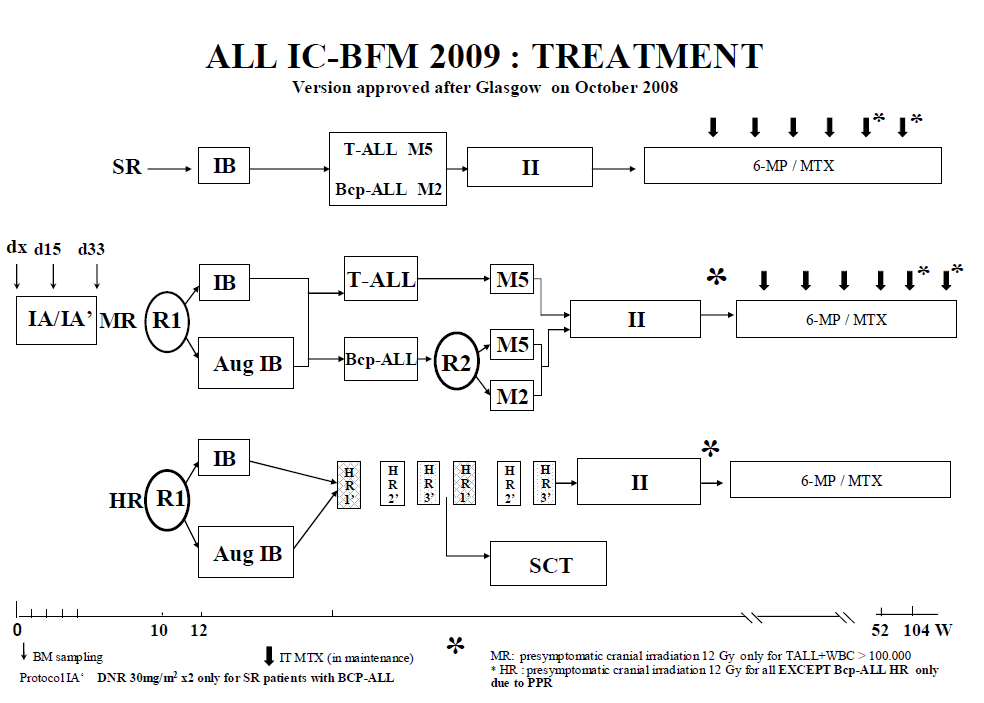


Figure S1. The ALL treatment protocol scheme.
